# Academic Integrity Perceptions Among Health-Professions’ Students: A Cross-Sectional Study in The Middle East

**DOI:** 10.1007/s10805-022-09452-6

**Published:** 2022-07-05

**Authors:** Gomathi Kadayam Guruswami, Sabiha Mumtaz, Aji Gopakumar, Engila Khan, Fatima Abdullah, Sanjai K. Parahoo

**Affiliations:** 1 Department of Biomedical Sciences, Gulf Medical University, Ajman, UAE; 2 University of Wollongong in Dubai, Dubai, UAE; 3 Lecturer-Statistics, Gulf Medical University, Ajman, UAE; 4 Student, Bachelor of Biomedical Sciences Program, Gulf Medical University, Ajman, UAE; 5 Program Chair, School of Business and Quality Management, Hamdan Bin Mohammed Smart University, Dubai, UAE

**Keywords:** Academic dishonesty, Academic integrity, Cheating, Health-professions’ students, Plagiarism, Medical students

## Abstract

A high level of professional integrity is expected from healthcare professionals, and literature suggests a relationship between unethical behavior of healthcare professionals and poor academic integrity behavior at medical school. While academic integrity is well researched in western countries, it is not so in the Middle East, which is characterized by different cultural values that may influence students’ academic integrity conduct. We conducted a cross-sectional study among health-professions students at a university in the Middle East to assess perceptual differences on various cheating behaviors, as well as to explore the reasons underlying the cheating behavior. A validated survey instrument disseminated among first and second-year undergraduate students resulted in 211 complete responses and this data was analyzed using descriptive and inferential statistics. Pearson’s Chi-square/ Fischer’s exact test was applied to test the association of various factors with academic misconduct. The major determinants of academic misconduct were investigated using Binary Logistic regression model. The conducted analysis and the results showed that preceding cheating behavior was the only factor significantly associated with cheating in the university (p < 0.001). No association was found between cheating behavior and age, college/major, awareness regarding academic integrity, or perception of faculty response. The reasons provided by students for cheating behavior were mainly academic workload and pressure to get a good grade. Various suggestions are made to enhance academic integrity among health-professions students including organizing workshops and events by the university to increase awareness and create an academic integrity culture, providing peer guidance as well as emotional and social support.

**Supplementary information:**

The online version contains supplementary material available at 10.1007/s10805-022-09452-6.

## Introduction

Academic integrity (AI) is defined as a commitment to the fundamental principles and values - honesty, trust, fairness, respect, responsibility in learning, teaching, and research, and courage (Bretag, 2016; International Center for Academic Integrity, 2021; Universities Australia, 2017). These principles and values present a foundation for guiding the behavior of students and other members of the academic community and translating written norms and ideals into practice.

AI is fundamental in education and is the foundation in preparing students to be successful, both personally and professionally (Fishman, 2013). Although adherence to AI principles and core values is crucial for all disciplines, it is particularly significant for health-professions students because any lapses could compromise patients’ safety (Korn & Davidovitch, 2016). In addition to having adequate clinical skills, students from health-professions are expected to behave in accordance with the highest ethical and professional standards to qualify as competent medical professionals who can provide proper healthcare for patients (LaDuke, 2013; Yadav et al., 2019). However, available literature suggests that unethical health-professionals’ behavior may be influenced by poor integrity behavior and absence of adherence to the AI and ethics principles and values during the education in medical school (Baxter & Boblin, 2007; LaDuke, 2013).

While there is agreement among the medical community that medical ethics education of health-professions students promotes professionalism in the workplace leading to its wide adoption by medical schools (Carrese et al., 2015; Rabow et al., 2010), there is less emphasis on AI and general ethical behavior (Younis & Gishen, 2019). Many practicing physicians and medical students have expressed dissatisfaction with the lack of emphasis on general ethics education during medical school (Carrese et al., 2015) and it is critical to increase this emphasis during medical training in order to develop ethical patient-doctor relationships (Rios, 2016). Education has a considerable influence on a person’s attitude and behavior, and significant changes in students’ ethical beliefs have been observed as they progress through medical school (Price et al., 1998). More recently, Ballantine et al., (2018) proposed teaching students how to address workplace ethical dilemmas in order for them to act more ethically in the workplace.

Further, the impact of student academic dishonesty is not just limited to educational institutions, but it also has a correlation with unethical behaviors in workplaces (Ballantine et al., 2018; Harvey et al., 2020; LaDuke, 2013) and corruption in countries (Teixeira, 2013). It has been found that cheating during studies was followed by manipulation of clinical data, such as recording patients’ vital signs that were not taken or medicines that were never administered (Baxter & Boblin, 2007).

Therefore, a worldwide increase in disciplinary measures is being witnessed by the medical boards against doctors who engage in unprofessional, incompetent, or improper medical behavior (Papadakis et al., 2005). United States statistics from 2017 showed 8,813 medical state board actions leading to various disciplinary actions, including 264 revoked licenses (U.S. Medical Regulatory Trends and Actions, 2018) and DuBois et al., (2019) found many repeated instances of intentional wrongdoing in their analysis of 280 cases of serious ethical violations. Such unethical behavior is not limited to physicians but also observed in medical/biomedical research (Lancet, 2015).

Research integrity too like AI is critical to sustaining scientific excellence and public trust, as both are directed towards performing academic activities in a trustworthy and responsible manner. Scientists’ professional integrity is critical to society as a whole, and particularly to disciplines such as medicine, which rely heavily on scientific advances for advancement (Korenman et al., 1998). Researchers must commit to intellectual honesty when proposing, conducting, and reporting research, as well as accept responsibility for their actions and practices (National Research Council, 2002). However, falsification and fabrication of medical research data, as well as detrimental research practices are all too common. They may result in a waste of financial and human resources, and pose a risk to human health (Fanelli, 2009). For example, a few years ago, 42 papers submitted by medical researchers between 26-31March, 2015 had to be retracted (Lancet, 2015) and recently, two papers related to Covid-19 research were retracted by the authors since the primary data source could not be validated (Lancet, 2020, NEJM 2020). This is alarming since the fraudulent research practice directly translates into unreliable medical evidence and discovery, potentially endangering human lives.

Furthermore, while AI is well researched in western countries, there is a lack of relevant research in the Middle East (AlJurf et al., 2020; Craig & Dalton 2014), specifically studies among health-professions students (Abdulrahman et al., 2017; Al-Qahtani & Guraya, 2019; Elzubeir & Rizk, 2003; Sattar & Roff, 2016). Since distinct sets of cultural values and traits shape behavior in different situations, the study findings in the western environment cannot be immediately applied to other contexts such as the Middle East, as students belonging to high collectivistic cultures may perceive cheating as helping each other (Harvey et al., 2020; McCabe et al., 2008).

Against this backdrop, the objectives of this study may be stated as investigating perceptions of students relating to:

- their knowledge and level of understanding of AI issues,
- their awareness of university AI policies,
- their attitudes and behavior towards AI,
- the institutional and personal factors affecting AI.

To this end, a cross-sectional survey was conducted, at a leading health-professions university in the Middle East, to assess the AI perceptions of entry-level (year 1 & 2) health-professions students. The study findings are expected to contribute to the AI literature in the Middle East, educate the AI policy at the university, while providing a valuable benchmark to other health-professions schools in the region.

## Literature review

### Academic misconduct in health-professions majors

AI is a fundamental issue in education (Comas-Forgas & Sureda-Negre, 2010; Williams et al., 2014, McCabe & ICAI, 2015) including health-professions education as highlighted by various studies (Ewing et al., 2019; Harvey et al., 2020; LaDuke, 2013; Macale et al., 2017; Younis & Gishen, 2019). These studies cover a spectrum of health-professions majors e.g. 74% of students enrolled in pharmacy admitted to collaborating on an assignment meant to be done individually (Rabi et al., 2006); nursing students accepted their cheating habits as normal, as they progressed through their academic careers (Macale et al., 2017; LaDuke, 2013); focus group discussions with students at a medical school highlighted that many students found it acceptable to cheat on exams (Okoye et al., 2018); and doctoral health science students did not give due importance to plagiarism (Ewing et al., 2019). Furthermore, students and teachers in the health-profession have a long-held belief that clinical misconduct is more serious than academic dishonesty (Keener et al., 2019). Existing literature thus highlights the pressing issue of academic dishonesty in the field of health-professions education, emphasizing the critical need for students to understand the implications and significance of AI (Younis & Gishen, 2019).

### AI in a multicultural milieu

Students have varying perspectives on what constitutes cheating and the relative gravity of various cheating practices depending on their cultural backgrounds (Ewing et al., 2019). The findings are in line with Williams et al., (2014), who found that fewer respondents from the Middle East perceived various academic misconduct behaviors as serious when compared to those from the United States. Guraya (2018) were also in alignment, not only did they find differences in perceptions of the seriousness of misconduct and recommended sanctions between students in medical schools in the UK and Saudi Arabia, but also between instructors from the two contexts, indicating that culture plays an important role.

These cultural differences may result in academically dishonest student behavior due to a lack of clear understanding of AI. In fact, Brown et al., (2018) identified this as a major reason for higher incidence of academic dishonesty among international students. Also, when Okoroafor et al., (2016) conducted a cross-national study to investigate the differences in levels of academic dishonesty self-reported by students, they found that Nigerian students engaged in more dishonest behavior than students from New Zealand due to cultural expectations, norms, beliefs, and values. This is consistent with Williams et al., (2014) findings, that some acts which are considered cheating in some countries, may be perceived as support gestures in others. For example, in collectivistic countries, students may perceive cheating as helping each other (Harvey et al., 2020; McCabe et al., 2008).

### Factors underlying students’ academic dishonesty

Extant literature has numerous studies attempting to investigate the reasons underlying students’ cheating behavior. Okoroafor et al., (2016), found that students’ engagement in academic misconduct could be related to (a) deontology, when students lack the knowledge of the academic regulation and what academic dishonesty means, and it also relates to the notion that students justify and define their actions as good or bad based on a set of personal, corporate, and religious rules without considering the consequences, (b) utilitarianism, when dishonest act is justified by focusing on the outcomes, not the process, (c) rational fair exchange, justifying dishonest act by assuming it publicizes the work of others, (d) Machiavellian perspectives, (e) cultural relativism and (f) situational factors including familial pressure or unforeseen circumstances. Further contributory reasons for committing academic dishonesty could be, poor time management skills, higher number of assignment submission requirements and easy access to information databases and communication technologies (Comas-Forgas & Sureda-Negre, 2010). Conversely, personality traits of conscientiousness and agreeableness were found to significantly reduce risk of cheating (Harvey et al., 2020). In addition to the previously listed reasons, the peer effect i.e. students’ perceptions of peer behaviour also has an influence on committing academic dishonesty (Carrell et al., 2011). Perceived acceptance of cheating behaviors among peers, reduced fear of getting caught and anticipated reactions of faculty to cheating also heavily influence academic dishonesty (Beasley, 2014; Ives & Giukin, 2020). More recently, Kiekkas et al., (2020) have succinctly grouped the reasons behind the academic dishonesty to three factors, “absence of severe consequences for cheating”, “the way examinations are performed”, and “the importance of achieving high grades”.

## Methods

To achieve the research objectives, a cross-sectional study was conducted at a leading co-educational health-professions university in the Middle East with a student population spanning across more than 80 different nationalities, with majority being from Gulf Co-operation Council (GCC) countries. Further, the students came from different academic systems (UAE Secondary School, American High School, British A-levels, International Baccalaureate, and the Indian High School), making the findings generic and independent of the any academic system.

A questionnaire was constructed using validated items for the study variables, sourced from the literature (Appendix 1). The questionnaire was discussed with three subject experts to establish face validity and it was modified based on their suggestions. Subsequently, the revised questionnaire was piloted with ten students to assess comprehension of the questions. The pilot test resulted in a few minor changes in item wordings, leading to the final questionnaire.

After securing ethical approval from the Ethics Committee of the university, an online cross-sectional survey was created using Google Forms using a built-in feature that allows respondents to submit only one response. Furthermore, the survey was emailed to the students on their college email id to ensure that only registered university students participated in the survey. Sampling frame consisted of the population of first-year and second-year health-professions students enrolled in various undergraduate programs which included Medicine, Dentistry, Pharmacy, Physiotherapy, Nursing, Biomedical Sciences, and Medical Laboratory Sciences. These students were still relatively new to the university and had no prior industrial experience that is normally obtained after getting relevant qualifications and through specializing and working in a given field. As a result, the students did not have exposure to professional deontological standards during their industrial training, as well as practical workplace experience, which might have shaped their perceptions of AI. Furthermore, surveying the whole population controlled for any bias in sampling. Participation in the study was voluntary and the cover letter assured the respondents of anonymity and confidentiality of their responses. Further, they were also informed that the data would be presented as group statistics and would not identify any individual respondent. The survey link was disseminated, through the office of student affairs, to the targeted population and class announcements were used to increase the survey awareness. Two reminders were sent at weekly intervals to increase the participation rate. The process resulted in 211 completed surveys which represented an adequate sample size for statistical analysis adopted in the study.

Descriptive and inferential techniques were adopted to analyze the data using IBM SPSS-25 software. Pearson’s Chi-square test or Fischer’s exact test was applied to test the association of various factors with level of academic misconduct among students. Major determinants of academic misconduct were identified using Binary Logistic regression model with a binary dependent variable titled ‘Level of academic misconduct’ (Never and Sometimes/Often/Very often), with level of significance set at p ≤ 0.05.

## Results

### Demographics

A total of 830 students were invited to take the survey and 211 students (25.4%) responded. There were no incomplete surveys, as submission was locked until after the students had responded to all questions. Table 1 shows the demographic characteristics of the respondents, which demonstrates that the majority (77%) were female, almost all (90%) were under the age of 20, and they were enrolled in various undergraduate programs, with over 44% being in Medicine. These statistics were compared with the target population statistics from the university factbook and found to be broadly similar thereby indicating that the sample was representative of the population, and it was subsequently used for further analysis.

**Table 1:** Demographic characteristics of the respondents and target population

Student Details		Respondents Count (%) (1st and 2nd year UG)	Target Population (All 1st & 2nd year UG)
Gender	Female	163 (77%)	70%
	Male	46 (22%)	30%
	Prefer not to say	2 (1%)	-
Age	17–20 years	189 (90%)	84%
	21–24 years	15 (7%)	9%
	Above 24 years	7 (3%)	7%
Major	Medicine	93 (44%)	39%
	Allied Health	60 (28%)	28%
	Dentistry	34 (16%)	17%
	Pharmacy	13 (6%)	10%
	Nursing	9 (4%)	4%
	Healthcare management	2 (1%)	2%
Total (count)		211	830

The rest of this section is structured using the framework of the four research objectives (RO).

### RO 1: students’ knowledge and level of understanding of AI issues

#### When did students first learn about AI?

**Table 2:** Students’ first learning about AI

First learned about AI	Number	Percentage
College	26	12%
High School	79	38%
Middle school	74	35%
Don’t know about it	32	15%

As shown in Table 2, most students (73%) reported having first learnt about AI at either middle or high school, thereby indicating they were aware of AI issues prior to enrolling at the university.

#### How did students learn about AI at the university?

**Table 3:** Students’ source of information about AI at the university

Source of information	Number	Percentage
At program orientation	104	49%
From the faculty	96	45%
At the white coat ceremony	68	32%
Through student handbook	60	28%
During the library orientation	30	14%
Others	11	5%
**Total**	211	

Multiple responses were permitted for this question (see Table 3). Results suggest that students’ primary source of information about AI, at the university, was program orientation (49%); followed closely by faculty communications (45%) and white coat ceremony (32%). While having a number of sources to communicate information about an important topic such as AI is inherently beneficial, there is room for improvement in the form of a more effective integrative communication strategy. As indicated by the findings (see Table 3), each information source individually reached less than half the students, therefore failing in the purpose of wider dissemination.

#### Students’ level of understanding of AI

**Table 4:** Students’ understanding of AI

How do you rate your understanding of AI	Number	Percentage
Very good	58	28%
Good	116	54%
Fair	31	15%
Poor	6	3%

Most students (82%) reported a good or very good level of understanding AI, while only 3% reported a poor level (see Table 4). While the responses might incorporate some social desirability bias, nonetheless, the findings demonstrate that students had a strong appreciation of AI issues, particularly that the majority learnt about AI in school, prior to joining the university (see Table 2).

### RO 2: students’ awareness of university AI policies

**Table 5:** Students’ awareness of University AI Policies

Does the university have an AI policy?	Number	Percentage
Yes	114	54%
No	17	8%
Don’t know	80	38%

The responses were mixed with just over half of students (54%) correctly reporting that the university had an AI policy while a substantial number (38%) were not aware of the University having such a policy (see Table 5). This further reinforces above discussion (see Table 3) highlighting the need for a more effective integrative communication strategy.

### RO 3: students’ attitudes and behavior towards AI

#### Students’ assessment of the AI policy at the university

**Table 6:** Assessment of the AI policy at the university

Assessment of the AI policy at the university	Number	Percentage
Strong	35	17%
Moderate	117	55%
Mild/Weak	59	28%

While 17% of students assessed the AI policy as “Strong”, the response may be considered as mixed, with just over half the students (55%) rating the university AI policy as “Moderate” (see Table 6). This may indicate that the policy in place was indeed perceived as moderate. Alternatively, viewed in conjunction with Table 5 where only 54% of students reported being aware of the AI policy, many students may have been unsure and thus chose the central scale point. This supplements previous findings (see Tables 3 and 5) indicating the need for better communication about the university AI policy and also more effective implementation.

#### Students’ perception about seriousness of types of academic misconduct

**Table 7:** Students’ perception about seriousness of academic misconduct behaviors

Academic misconduct behaviors	Not serious	Serious*	Percentage of Students rating behavior as serious
Cheating using notes in an exam/test/quiz	21	190	90%
Copying from another student in an exam	23	188	89%
Giving another student answer during an exam	20	191	91%
Doing an assignment for another student	43	168	80%
Copying an assignment from another student	36	175	83%
Getting your assignment done by another student	36	175	83%
Paying someone to do your assignment	30	181	86%
Copying material from websites and passing it off as your own	32	179	85%

*Serious aggregates responses to ‘somewhat serious’; ‘serious’ & ‘very serious’

As shown in Table 7, top three academic misconduct behaviors perceived as serious by the students were: giving another student answer during an exam (91%); cheating using notes in an exam/test/quiz (90%); and copying from another student in an exam (89%). This shows that students considered academic dishonesty linked to exams as more serious than misconduct related to assignments.

#### Students support for AI policy

**Table 8:** Students support for AI policy

How would you describe student response/support for academic dishonesty?	Number	Percentage
They don’t support it	7	3%
They expect to be let off with just warning	59	28%
They support it for what they perceive as severe violations	85	41%
They support it totally	60	28%

Majority of students (69%) either fully supported university AI policy or did so in instances of severe violations, whereas 28% students expected the university to be lenient and be let off with a warning (see Table 8).

#### Students’ suggestions for addressing academic dishonesty at the university

**Table 9:** Students’ suggestions for addressing academic dishonesty at the university

Student Responses	Number	Percentage
Increase awareness through peer discussions and workshops	134	64%
Provide online resources with tips and information to avoid plagiarism	54	26%
Stricter penalty and implementation by faculty	53	25%
Others	15	7%

Students were given the choice to select more than one option as well as provide open responses (see Table 9). A vast majority of students (64%) felt academic dishonesty should be addressed by increasing awareness through workshops and peer discussions, followed by the need for more resources and tips for avoiding plagiarism (26%). This was an important finding since the student recommendations were skewed towards proactive and supportive measures, as opposed to punitive interventions, in alignment with Stephenson & Roff (2015) findings in a medical school in London. Other suggestions by students included reducing the workload, providing counselling for the students and stricter invigilation during examinations.

### RO4: Institutional and personal factors affecting AI

#### Association of various factors with cheating behavior

Students’ engagement in academic misconduct was investigated by asking *“How often have you engaged in this behavior*” using a 4-point Likert scale (Never, Sometimes, Often, Very Often), for the eight academic misconduct behaviors enumerated in Tables 7 and scored from 1 to 4. To find the association between self-reported misconduct and listed factors, ‘sometimes, often and very often’ were clubbed together as indicator of engaging in misconduct and ‘never’ was kept separate to indicate no misconduct.

Potential associations between demographics and students’ cheating behavior were investigated using Pearson’s chi-square test. No significant association was found between age of the students (p = 0.36), gender (p = 0.94), college or the program that the students were enrolled in (p = 0.07), and level of awareness regarding AI (p = 0.09).

As shown in Table 10, perception of faculty response to cheating behavior (p = 0.001) and previous academic misconduct (p < 0.001) were the only factors found to be significantly associated with students’ cheating behavior.

**Table 10:** Factors associated with students’ academic misconduct

Factor		Self-reported academic misconduct in university				P value*
		**Never**		**Sometimes/Often/** **Very often**		
		**Number**	**%**	**Number**	**%**	
**Student perceptions of seriousness of academic misconduct**	Not serious	30	44.8%	37	55.2%	p = 0.09 (NS)
	Serious	82	56.9%	62	43.1%	
**Previous cheating behavior**	Never	65	95.6%	3	4.4%	P ≤ 0.001
	Sometimes/ Often/very often	47	32.9%	96	67.1%	
**Student perceptions of knowledge about AI**	Poor knowledge	15	40.5%	22	59.5%	p = 0.09 (NS)
	Good knowledge	97	55.7%	77	44.3%	
**Student perceptions of faculty response to academic dishonesty**	Mild/weak (warning with no action)	14	38.9%	22	61.1%	p = 0.001
	Moderate (they take action, but it is not too strict)	45	45.0%	55	55.0%	
	Severe (they take strict action)	53	70.7%	22	29.3%	

*Pearson’s chi-square test of association used, p ≤ 0.05*was taken as significant, NS- Not significant*

#### Assessing the major determinant of academic misconduct in the University using logistic regression

The above mentioned two significant factors were further examined using binary logistic regression, and only one factor, namely students’ previous cheating behavior, was found to be associated with the academic misconduct (see Table 11).

**Table 11:** Major determinant(s) of academic misconduct in the university^a^

Determinant	Regression coefficient	S.E.	P value	Adjusted OR*	95% CI for Adjusted OR	
					Lower	Upper
Previous misconduct experience	3.76	0.62	0.000*	42.9	12.8	144.2
Faculty response to misconduct	0.30	0.44	0.49	1.4	0.6	3.2

^a^Degree of association by binary logistic regression model; *significant

#### Cheating behavior of students before and after coming to the university

Since students’ previous misconduct experience was a major determinant of academic misconduct in the university, it was further investigated to see if the cheating behavior of students had altered after coming to the university (see Table 12). As shown in Table 12, the number of students reporting never cheating has gone up from 32 to 53% indicating lesser numbers are indulging in cheating behavior after coming to the university, providing support to the effectiveness of university AI policies. Wilcoxon Signed-rank test also indicated significantly lesser cheating behavior after coming to the university (Median score = 8, IQR = 3) compared to before joining university (Median score = 10, IQR = 5), p < 0.001.

**Table 12:** Students’ cheating behavior before and after coming to the university

Item	*Number of students*	*Percentage*
***How often have you cheated BEFORE coming to the university?***		
Never (Score 8)	68	32.2
Sometimes/Often/very often (Scores 9–32)	143	67.8
***How often have you cheated AFTER coming to the university?***		
Never (Score 8)	112	53.1
Sometimes/Often/very often (Scores 9–32)	99	46.9

#### Students’ perception of faculty response to AI violations

**Table 13:** Students’ perception of faculty response to AI violations

Faculty response to AI violations	Number	Percentage
Severe, they take strict action	75	36%
Moderate, they take action, but it is not too strict	100	47%
Mild/weak, they warn and let you off	36	17%

The responses show that majority of the students (64%) perceived the faculty response to AI violations, to be moderate or weak. Hence, there is scope for more decisive instructor actions to send a stronger message to the students and reduce AI violations (see Table 13).

#### Underlying reasons for academic dishonesty behavior

**Table 14:** Reasons for Academic Dishonesty Behavior, as perceived by the students

Reasons	Number	Percentage
Lack of time to do the assignments/study	137	65%
Pressure to get a better grade	132	63%
Peer Pressure to help others	114	54%
Others are also doing it, so why should I not?	63	30%
Low chance of being caught	73	35%
Low penalty even if caught	66	31%
Been doing it from school	68	32%
Do not know it is wrong to do so	49	23%

As shown in Table 14, the three major causes (multiple options could be selected) for academic dishonesty behavior reported by the students were lack of time to do the assignments/study (65%); pressure to get a better grade (63%); and peer pressure to help others (54%).

#### Frequency of faculty reference to AI policy during the semester

**Table 15:** Number of times the faculty referred to AI policy during the semester

Frequency of references to the AI/dishonesty in a semester	Number	Percentage
Never	72	34%
Once in a semester	71	34%
Once in a month	44	21%
Once in a week	17	8%
More than once a week	7	3%

The responses showed that majority of the faculty (68%) rarely or never referred to AI policies during the semester (see Table 15). These are serious findings and highlight the need to address this issue through faculty training and regular communication with faculty to ensure their support and commitment in disseminating the relevance and importance of AI to the students.

## Discussion

The current study has contributed to the AI literature by uncovering several interesting findings, that add to our current understanding of health-professions students’ knowledge and awareness of AI issues, their attitudes and behaviors toward AI, and their perceptions of the various institutional and personal factors that contribute to AI.

An analysis of student perceptions towards different types of cheating behavior demonstrated that all acts of cheating were not considered as equally serious by the students, with exams related misconduct being construed as more serious than assignment-related cheating incidents. In addition, “copying an assignment from another student” was considered more serious than “doing an assignment for another student”, implying that students assign seriousness in cheating behaviors to accountability, with the onus on the one asking for unauthorized help while absolving the one providing help. This may be explained according to Hofstede’s (2011) cultural dimensions. The university’s student population comes from a predominantly “collectivistic” culture with the majority of students coming from Arab and Asian countries. Within the collectivist society of the Middle East, a student who assists another student in cheating may even psychologically justify their own behavior as extending a helping hand rather than engaging in an act of cheating. Another explanation could be rooted in the gender or sex role socialization. Most of the respondents were female (77%) and according to (Whitley et al., 1999), females are generally more helpful and supportive displaying enhanced collectivistic traits. However, no association was seen between gender and cheating behavior as has also been reported by others (Ip et al., 2018; Özcan et al., 2019).

An encouraging finding is that the majority of students expressed support for the university AI policy, at least in instances of severe violations. Furthermore, students recognized that “paying to get assignments done” (i.e., contract cheating) was a serious form of cheating, indicating an awareness of contract cheating. However, it appears that the external contracting aspect, rather than the fact that the assignment is being done by someone, is creating this unease in the students’ mind. This is inferred from the finding, which shows a sharp decrease in the percentage of students who consider copying an assignment or having it done by another student to be “very serious”. This could be related to the nature of their behavior in a collectivist milieu. It is also pertinent that the percentage who did not cheat after joining the university was higher than those reporting having cheated before joining the university. This would imply that the university AI environment is supporting a change in behavior among the students towards AI.

Why, then, do some students still choose to cheat? The students mentioned three major reasons for engaging in cheating behavior: lack of time to do the assignments/study; pressure to get a better grade; and peer pressure to help others. These reasons have also been reported by others (Carrell et al., 2011; Comas-Forgas & Sureda-Negre, 2010; Ip et al., 2016). While the first reason pertains to time management and is a personal issue, the next two causes pertain to cultural factors relating to societal expectations and collectivism cultural traits.

What practical recommendations for university management to derive from the findings? In developing strategies to reduce academic misconduct, it is important to understand its antecedents. In this regard, a single factor, students’ previous cheating behavior, was found to be associated with the academic misconduct, in alignment with Ip et al., (2016) findings. Age and gender of the students, the academic major, and their level of awareness regarding AI were not found to be associated with cheating behavior. As a result, it is critical that the university places a strong emphasis on AI and establishes an environment that discourages academic misconduct and socializes entry-level students with strong AI values, despite their varying educational backgrounds and prior experiences.

Creating such an environment necessitates the dissemination of consistent messages by all academics and administrators via an integrated communication strategy (Eury & Trevino, 2019). This also addresses the findings that highlight insufficient student reach (less than 50%) with each piecemeal individual source in isolation (Table 3). Student orientation could be the starting point for this communication, as the majority of students said this was the first time they heard about AI at the university. However, it should be a consistent and continuous process to keep message in recent memory of the students as well as emphasize the importance of AI. This is supported by findings showing poor recall rate, with 46% students not aware of university AI policy (Table 5). A key reason for this could be the faculty’s inconsistent and irregular informational reinforcement, with nearly three-quarters of faculty (68%) referring to AI policies only once or twice during the semester (Table 15). Therefore, it is recommended that instructors should be explicitly advised to refer to the AI requirements frequently during the course of a semester. It may be especially beneficial to emphasize the significance of AI and the university’s AI policy prior to assessment submissions and examination. This is in line with Morris (2018) findings that developing the AI strategy, reviewing related policies and practices, and faculty professional development, were key to promoting AI.

Developing and maintaining a culture of AI remains a challenge for many universities, particularly in the digital era, where access to information and unauthorized assistance is easy and only a click away. More needs to be done to help students better understand the importance of ethical behavior and develop a moral contract to guide such behavior, leading to professionalism in future workplaces (Keefer et al., 2020). Simultaneously, there is a need to develop student support systems that will both support students and prevent them from engaging in academically dishonest behavior.

A practical way to accomplish this would be to form student and faculty groups that would champion AI by sensitizing and guiding peers to the University’s AI policies and other AI-related resources. These AI champions could also contribute to the creation of engaging AI content by providing examples in the form of case scenarios, which could then be converted into an interactive online format and shared with all students via the learning platform. Furthermore, the university may organize workshops and events to create an AI culture. Social media also represents an effective channel to reach out to the students and engage them. This strategy would also be in line with student recommendations for reducing academic misconduct, which were centered on proactive and supporting measures, rather than punitive tactics. In addition, interventions such as promoting more student/formal exchanges, providing peer guidance, and dialogue between different cultures may also be valuable while tackling issues related to academic misconduct (Okoroafor et al., 2016).

Finally, the university could also assist students by providing a safe space for them to express their concerns about academic workload and other challenges. This would provide much-needed emotional and social support in order to promote inclusivity and combat peer pressure. Furthermore, a strong yet consistent and fair application of academic misconduct sanctions will create and sustain a culture of AI, as well as develop ethical values and professionalism in health-professions students.

## Conclusions

The present study has unearthed the perceptions of students relating to: their level of understanding of AI issues, their awareness of university AI policies; their attitudes and behavior towards AI; and finally, the institutional and personal factors affecting AI. It was determined that the only antecedent of cheating behavior was preceding cheating behavior. Furthermore, it was interesting to note that a lower percentage of students reported to have cheated since joining the university as compared to during their secondary schooling. This would indicate that the efforts of the university towards creating a conducive AI environment have been fruitful. However more needs to be done, particularly as more universities adopt a blended learning environment. These strategies revolve around integrated communications to increase awareness and develop positive attitudes towards AI, developing a positive university culture for AI, and supporting students in the process of adopting ethical behaviors.

## Study limitations

A limitation of our study is that it explores self-reported perceptions and behavior, thus introducing the possibility of social desirability bias. This was controlled to some extent through survey anonymity and clear communication of the same to the respondents. Further, since the study was anonymous, it was not possible to follow up students to ensure greater participation and improve the response rate from 25.4%. However, we do feel that our study has a representative sample of our student population, as per the reported statistics from the university fact-book. Finally, the study has been conducted in a single university context in the Middle East, and although the demographic profiles of students in other regional universities would not be dissimilar, any extension of findings to external contexts should be done with the usual caution.

## Electronic supplementary material

Below is the link to the electronic supplementary material.

Supplementary Material 1

## Data Availability

All data will be made available on request.
