# Supplementary material for: Academic Integrity Perceptions Among Health-Professions’ Students: A Cross-Sectional Study in The Middle East
Source: J Acad Ethics. 2022 Jul 5;21(2):231–49. doi: 10.1007/s10805-022-09452-6 (PMC9255445; doi:10.1007/s10805-022-09452-6)
Supplement: Supplementary file 1 — Supplementary Material 1 [file 10805_2022_9452_MOESM1_ESM.docx]

**Appendix 1 - Survey questionnaire**

The survey included questions used by previous researchers, as well as some developed for the current study. Some questions were modified to fit the research objectives.

| **Question** | **Reference** |
| --- | --- |
| **Demographic characteristics**  (gender, age, and college) |  |
| **When did you first learn about academic integrity?**  (Middle school/High School/College/Don't know about it) | (Bretag et al., 2014) |
| **How did you learn about academic integrity in GMU? (Tick all that apply)**  (At program orientation/From the faculty/At the white coat ceremony/Through student handbook/During the library orientation/Others) | (Academic Honor Policy Committee, 2015) |
| **How do you rate your understanding of academic integrity?**  (very good/good/fair/poor) | (Bretag et al., 2014; Simon et al., 2004) |
| **Does GMU have an Academic Integrity policy? (It can also be called the Academic dishonesty or cheating policy)**  (Yes/No/Don’t know) | (Tabsh et al., 2017) |
| **How would you rate the Academic integrity policy at GMU?**  (Strong/Moderate/Weak) | (Academic Honor Policy Committee,2015) |
| **How serious do you rate the following academic misconduct?**  **(Rate as Not serious, somewhat serious, Serious, Very serious)**  (Cheating using notes in an exam-test-quiz / Copying from another student in an exam / Giving another student answer during an exam / Doing an assignment for another student / Copying an assignment from another student / Getting your assignment done by another student / Paying someone to do your assignment / Copying material from websites and passing it off as your own) | (Simon et al., 2004; Sutton & Huba, 1995) |
| **How do you think academic dishonesty should be addressed in GMU? (Tick all that apply)**  (Increase awareness through peer discussions and workshops / Provide online resources with tips and information to avoid plagiarism / Stricter penalty and implementation by faculty / Others) | (Tabsh et al., 2017) |
| **How would you describe student response/support for academic dishonesty?**  (They don’t support it / They expect to be let off with just warning / They support it for what they perceive as severe violations / They support it totally) | (Academic Honor Policy Committee,2015) |
| **How often have you seen this happen at GMU? (Rate as Never, sometimes, often, very often)**  (Copying an assignment from another student / Doing an assignment for another student / Getting your assignment done by another student / Copying material from websites and passing it off as your own / Copying from another student in an exam / Giving another student answers during an exam / Cheating using notes in an exam-test-quiz /  Paying someone to do your assignment) | (Academic Honor Policy Committee,2015) |
| **How often have you engaged in this behavior before and after coming to GMU?**  **(Rate as Never, sometimes, often, very often)**  Copying an assignment from another student / Copying from another student in an exam / Cheating using notes in an exam-test-quiz / Doing an assignment for another student / Giving another student answers during an exam / Getting your assignment done by another student / Copying material from websites and passing it off as your own / Paying someone to do your assignment) | To ascertain students’ engagement in academic dishonesty behavior before and after joining GMU, 8 statements were developed describing different academic dishonesty acts.  Some of the statements were modified from other studies e.g. Owunwanne et al., (2010) |
| **How would you rate faculty response to academic dishonesty?**  (Severe, they take strict action / Moderate, they take action but its not too strict / Mild/week, they warn and let you off) | (Tabsh et al., 2017; von Dran, 2001) |
| **Indicate your agreement regarding the following as possible reasons for academic dishonesty behavior at GMU**  (Lack of time to do the assignments-study / Pressure to get a better grade / Peer Pressure to help others / Others are also doing it, so why should I not? / Low chance of being caught / Low penalty even if caught / Been doing it from school / Do not know it is wrong to do so) | (Kwong et al., 2010; Tabsh et al., 2017) |
| **How often do you come across references to the academic integrity/dishonesty in a semester?**  (Never / Once in a semester / Once in a month / Once in a week / More than once a week) | (Tabsh et al., 2017) |

**References:**

Academic Honor Policy Committee. (2015). Academic Integrity Survey. Florida State University. Retrieved from: <https://fda.fsu.edu/sites/g/files/imported/storage/original/application/ba4467157e26476a3b47dfc6448cb24b.pdf>

Bretag, T., Mahmud, S., Wallace, M., Walker, R., McGowan, U., East, J., Green, M., Partridge, L., & James, C. (2014). “Teach us how to do it properly!” An Australian academic integrity student survey. Studies in Higher Education, 39(7), 1150–1169. https://doi.org/10.1080/03075079.2013.777406

Kwong, T., Ng, H. M., Kai-Pan, M., & Wong, E. (2010). Students’ and faculty’s perception of academic integrity in Hong Kong. Campus-Wide Information Systems, 27(5), 341–355. https://doi.org/10.1108/10650741011087766

Owunwanne, D., Rustagi, N., & Dada, R. (2010). Students’ Perceptions Of Cheating And Plagiarism In Higher Institutions. Journal of College Teaching & Learning, 7(11), 59–68. https://doi.org/10.4018/978-1-5225-1610-1.ch012

Simon, C. A., Carr, J. R., McCullough, S. M., Morgan, S. J., Oleson, T., & Ressel, M. (2004). Gender, student perceptions, institutional commitments and academic dishonesty: Who reports in academic dishonesty cases? Assessment and Evaluation in Higher Education, 29(1), 75–90. https://doi.org/10.1080/0260293032000158171

Sutton, E., & Huba, M. (1995). Undergraduate Student Perceptions of Academic Dishonesty as a Function of Ethnicity and Religious Participation. NASPA Journal, 33(1), 19–34.

Tabsh, S. W., Abdelfatah, A. S., & El Kadi, H. A. (2017). Engineering students and faculty perceptions of academic dishonesty. Quality Assurance in Education, 25(4), 378–393. https://doi.org/10.1108/mbe.2000.26704caa.006

von Dran, G. (2001). Can Students’ Academic Integrity Be Improved? Attitudes and Behaviors Before and After Implementation of an Academic Integrity Policy. Teaching Business Ethics, 5(1), 35–58. https://doi.org/10.1023/A:1026551002367
